# Live imaging of vascular dynamics in mouse skin

**DOI:** 10.1016/j.jid.2025.12.031

**Published:** 2026-02-12

**Authors:** Ino Song, Matthew Aaron Blumberg, Chen Yuan Kam

**Affiliations:** 1 Division of Dermatology, Department of Medicine, David Geffen School of Medicine, University of California, Los Angeles, Los Angeles, California, USA; 2 Eli and Edythe Broad Center for Regenerative Medicine and Stem Cell Biology, University of California, Los Angeles, California, USA

**Keywords:** Cutaneous vasculature, Endothelial cells, Intravital, Longitudinal, Methods and techniques for skin research, Multiphoton

## Abstract

The cutaneous vascular network, located in the skin dermis, plays vital roles in thermoregulation, immune surveillance, and tissue regeneration. Despite its important functions, the cutaneous vasculature remains an understudied component of the skin. This article outlines approaches for intravital 2-photon microscopy to visualize and track vascular dynamics in mouse skin, particularly the hind paw, which offers a simplified, hairless model for longitudinal imaging. Utilizing genetically encoded fluorescent reporters, endothelial cells can be selectively and dynamically labeled to study vascular remodeling events that occur over the time frame of days to weeks, necessitating longitudinal approaches to capture the dynamics and resolution of these processes. Mosaic labeling approaches enable the tracking of single cells within the context of the remodeling network, allowing for an understanding of cellular behaviors that drive remodeling events. Intravital imaging also provides an opportunity to visualize and measure vascular function such as hemodynamics and barrier function. Collectively, these techniques provide powerful tools to dissect postnatal vascular development, adult homeostatic maintenance, and response to injury and holds potential for the interrogation of fundamental aspects of vascular biology as well as the understanding of cutaneous pathologies that involve the vasculature.

## INTRODUCTION

The skin is a highly regenerative and dynamic organ that functions as a critical barrier to shield the body from the external environment (Fukuda et al, 2025). As such, it is of no surprise that the skin is a highly vascularized organ that contains an extensive vascular network that supports the metabolic demand of its consistently cycling populations of stem cells (Braverman, 2000). An emergent theme in the field of vascular biology is the organotypic specialization of vascular beds to the architecture and function of the organ in question (Augustin and Koh, 2017). The skin is no exception to this principle as the cutaneous vasculature is responsible for essential functions of the skin such as thermoregulation and immune surveillance, in addition to its roles in regulating blood—tissue exchange (Charkoudian, 2003; Egbuniwe et al, 2015; Link et al, 2024). Despite its importance, the cutaneous vasculature remains to be an understudied component of the skin. Given its external location on the body, the cutaneous vasculature is a uniquely accessible vascular bed that is amenable to noninvasive imaging approaches. A detailed protocol for intravital longitudinal imaging of mouse skin has previously been published, the principles of which can be readily applied to the imaging of the cutaneous vasculature (Pineda et al, 2015). This article aims to provide guidance on best practices and potential applications for longitudinal imaging of the cutaneous vasculature on the basis of insights that we and others have gained from studying this dynamic vascular network.

## TWO-PHOTON MICROSCOPY

In traditional single-photon microscopy, a single photon at a specific wavelength excites a fluorophore from a ground state to an excited state, and fluorescence signal is collected at a higher emission wavelength due to the Stokes shift (Luu et al, 2024). In contrast, 2-photon microscopy involves the simultaneous absorption of 2 photons of lower energy to excite a fluorophore, which then emits light of a shorter wavelength within the typical range of emission spectra utilized in single-photon microscopy (Luu et al, 2024; Xu et al, 2024). This imaging modality confers several important advantages that make it ideal for intravital imaging. First, the long wavelengths used for excitation allow for deep tissue penetration owing to lower levels of scattering and absorption by the tissue than shorter wavelengths (Xu et al, 2024). Furthermore, because simultaneous absorption of multiple photons occurs only at the focal point of a high-intensity pulsed laser, 2-photon imaging results in lower levels of phototoxicity (Luu et al, 2024). As such, 2-photon microscopy represents the ideal imaging technique for imaging live cells in mammalian tissues (Xu et al, 2024). Through this imaging modality, many impactful discoveries have been made, shining light upon the regulation of fundamental biological processes within the context of the native tissue environment of organs such as the brain, bone marrow, kidney, and intestine (Azkanaz et al, 2022; Hartmann et al, 2021; Ritsma et al, 2012; Sipkins et al, 2005). In recent years, the skin has emerged as a valuable and practical model for noninvasive live imaging purposes owing to its unique accessibility and diverse milieu of cell types and processes that can be readily visualized and longitudinally tracked over extended periods of time (Cockburn et al, 2022; Marsh et al, 2018; Sarate et al, 2024; Schoofs et al, 2025; Zhang et al, 2021).

## INTRAVITAL IMAGING OF MOUSE SKIN

**Figure 1.**
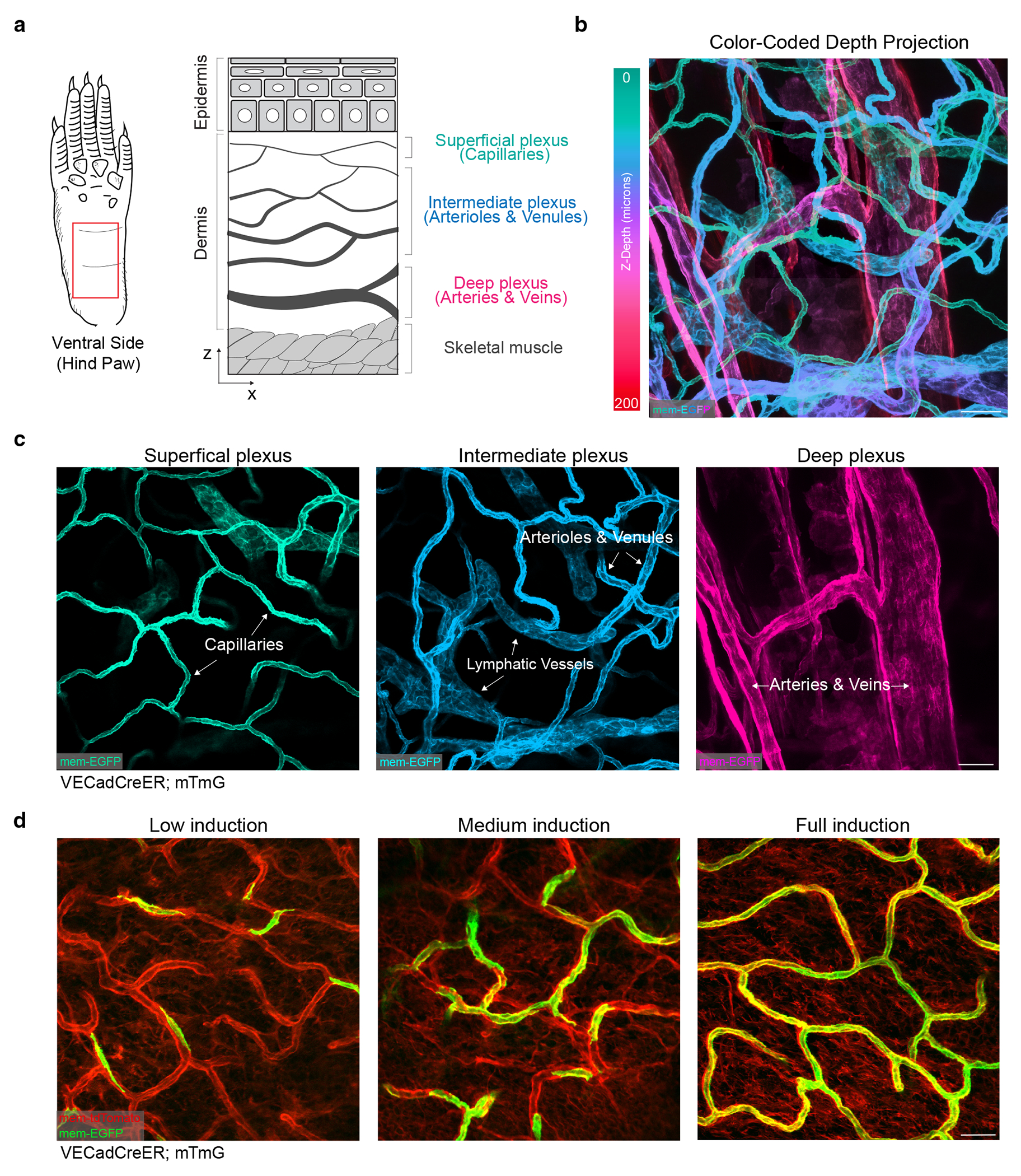
Anatomy of the murine palmoplantar skin vascular network. (**a**) Schematic of the ventral side of the mouse hind paw and cross-sectional schematic of the skin vascular network. The hairless region utilized for imaging is denoted by the red square. Skin vasculature resides in the dermis layer of the skin beneath the barrier forming epithelial cells of the stratified epidermis. (**b**) Color-coded depth projection of the skin vascular network. Fully recombined *VECadCreER; mTmG* was used to specifically label the vasculature of adult mice. Z-depth color code on the left designates the gradient from 0 to 200 μm below the epidermis. Bar = 50 μm. (**c**) Z-projections of the superficial, intermediate, and deep plexi. Bar = 50 μm. (**d**) Examples of low, medium, and full induction of tamoxifen in *VECadCreER; mTmG* adult animals allowing titratable expression of EC mem-EGFP. Bar = 40 μm (**a**, **b**, and **c**, adapted from Kam et al [2023] with permission from Elsevier). EC, endothelial cell.

Murine skin provides an ideal model for noninvasive intravital imaging because it is located on the outermost part of the body, allowing for the application of imaging approaches without the need for surgical exposure or implantation of imaging windows (Pineda et al, 2015). Mammalian skin is composed of 2 primary layers: the epidermis and the underlying dermis (Figure 1a) (Hsu and Fuchs, 2022). The epidermis is the outermost layer of the skin and forms a protective barrier over the body’s surface (Fukuda et al, 2025). It is made up primarily of specialized epithelial cells called keratinocytes that form a stratified squamous epithelium (Simpson et al, 2011). The dermis is located below the epidermis and is where the cutaneous vasculature is found along with various skin appendages such as hair follicles, sebaceous glands, and other components of the tissue stroma, which are embedded within a mesh of collagen extracellular matrix (Hsu and Fuchs, 2022).

The plantar skin of the mouse hind paw presents a simplified model of skin because it is devoid of hair follicles and, as such, does not require the removal of hair, while also avoiding the need to consider the timing of the hair cycle when performing experiments (Kam et al, 2023; Marsh et al, 2018; Wong et al, 2006). The hierarchical organization of the paw skin vasculature also confers unique advantages for the visualization, segmentation, and analysis of this 3-dimensional network (Kam et al, 2023). In this network, the vasculature is arranged in 3 horizontal plexi, whereby the superficial capillary plexus is located directly underneath the basal layer of epidermal stem cells (Figure 1a and b). Directly below the superficial plexus, an intermediate plexus consisting of arterioles, venules, and lymphatic vessels can be found (Figure 1c). Finally, the large caliber arteries and veins are located at the base of the dermis. These plexi can then be segmented by depth to generate Z-projections that enable the visualization and analysis of these plexi in the XY plane (Figure 1c).

## FLUORESCENT REPORTERS FOR IN VIVO VASCULAR IMAGING

To visualize the cutaneous vasculature, our laboratory mainly relies on genetically encoded fluorescent reporter lines for labeling vascular cells. For the purpose of this article, we will be focusing primarily on endothelial cells (ECs), which are the cells that line the inner surface of all blood vessels (Trimm and Red-Horse, 2023). These highly versatile squamous cells play essential roles for the regulation of vessel tone and permeability during homeostatic settings (Honkura et al, 2018). Furthermore, ECs play crucial cell-autonomous roles in the regulation of vascular remodeling in the context of development and regeneration (Trimm and Red-Horse, 2023). As such, substantial efforts have been made to understand the regulation and behaviors of ECs within the context of their native tissue environment. Although mural cells are known to play important roles for vascular function, this article will largely be focused on imaging tools and applications for studying EC biology.

Although there are various fluorescent reporter mouse lines that are suitable for live imaging purposes, we have found that the global double-fluorescent Cre reporter mouse, *mTmG*, enables exquisite labeling of vascular architecture and cellular morphology owing to its visualization of cell membranes (Muzumdar et al, 2007). In this reporter line, prior to Cre recombination, cell membrane—localized tdTomato (denoted as mem-tdTomato) expression is expressed ubiquitously. After Cre recombination, cells express membrane-localized EGFP (denoted as mem-EGFP), replacing the red fluorescence signal. To drive Cre recombinase expression ECs, there are several Cre driver lines that have been reported that would enable the specific labeling of ECs (Payne et al, 2018). We have utilized the tamoxifen inducible *VECadherinCreERT2* (*VECadCreER*) mouse line for our studies that is highly specific for ECs with no detectable leakiness within non—vascular endothelial-cadherin expressing cells (Sörensen et al, 2009). Note that this line is also known as *Cdh5CreERT2*. An important factor concerning the cell types that are labeled by this reporter line is that it drives expression in both blood and lymphatic ECs, therefore leading to labeling of both the blood and lymphatic vessel networks. Blood and lymphatic vessels can be distinguished on the basis of morphology whereby blood vessels possess consistent diameters along the length of vessels, whereas lymphatic vessels tend to have regions that are wide or narrow along their length. Skin lymphatic vessels also tend to be significantly wider than blood vessels in the intermediate plexus where the majority of the lymphatic network can be found (Figure 1c). Another significant advantage of the *VECadCreER; mTmG* model is that it allows for titratable induction of EC labeling on the basis of the dosage of tamoxifen applied. In this paper, we show an example of an adult *VECadCreER; mTmG* animal that has been treated with 10 μg (low induction), 100 μg (medium induction), or 2 mg (full induction) of tamoxifen through intraperitoneal (IP) injection, leading to increasing levels of EC mem-EGFP expression (Figure 1d). As such, this model can be used to induce mosaic single-cell labeling of ECs within the context of the larger vessel network for the tracking of EC morphology and behaviors at cellular resolution. This approach will be expanded upon in the following section.

## LONGITUDINAL TRACKING OF POSTNATAL VASCULAR DEVELOPMENT

**Figure 2.**
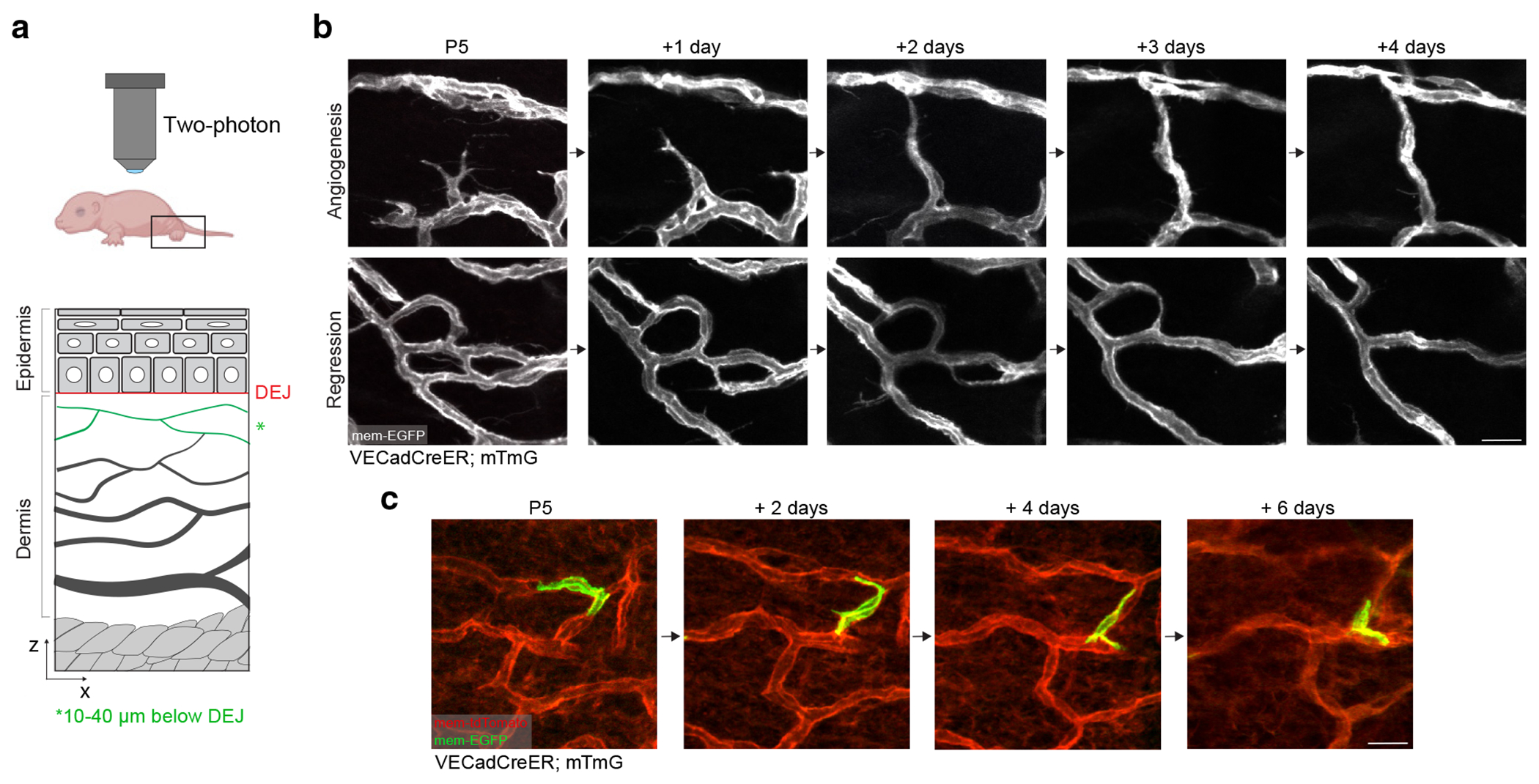
Longitudinal tracking of neonatal vascular remodeling and dynamic EC behavior. (**a**) Schematic of neonatal paw skin under investigation. The superficial capillary plexus is highlighted in green. It is located ~;5—30 μm below the DEJ. (**b**) Daily revisits of neonatal paw skin vasculature from P5 to P9. An example of an angiogenic sprout forming a new vessel connection is shown in the top panel. In the bottom panel, an example of sequential selective vessel regression is shown. Bar = 20 μm. (**c**) The luminal migration of a single labeled EC that is tracked from P5 to P11 with 2-day intervals between revisits. Bar = 20 μm (**a**, **b,** and **c**, adapted from Kam et al [2023], with permission from Elsevier). DEJ, dermal—epidermal junction; EC, endothelial cell; P11, postnatal day 11; P5, postnatal day 5; P9, postnatal day 9.

Neonatal paw skin presents a useful model for the longitudinal tracking of developmental vascular remodeling. From our previous studies, we have found that neonatal skin is a vascular network undergoing maturation, as longitudinal tracking of the skin capillary plexus over the course of postnatal development (postnatal day 5—postnatal day 15) revealed that vessel remodeling dynamics were largely driven by selective regression events compared with angiogenesis (Kam et al, 2023). Although less frequent than regression events, there are sufficient levels of angiogenic events occurring within this time frame to be a useful model for the tracking of the angiogenesis process. Daily revisits from postnatal day 5 to postnatal day 9 demonstrate the ability to resolve the stepwise progression of angiogenesis and pruning events (Figure 2b).

To induce labeling of ECs, it is important to ensure the proper delivery route of tamoxifen to neonates. As has been previously reported, we find intragastric (IG) injection to be the most efficient and effective method for neonatal administration of tamoxifen because IP injection of larger volumes is challenging owing to the small size of the IP cavity during neonatal stages (Pitulescu et al, 2010). For this procedure, we have found that a single IG injection of tamoxifen (100 μg) is sufficient to induce full labeling of the vasculature. Note that if the aim is to induce gene deletion through floxed alleles, subsequent daily administration of tamoxifen may be necessary depending on the efficiency of recombination of the alleles in question. To obtain sporadic labeling of ECs for single EC tracking (Figure 2c), we have utilized a dose of 2 μg of tamoxifen for sufficient numbers of labeled ECs while largely maintaining single-cell labeling throughout the entire skin vascular network. We typically perform IG injections at postnatal day 1 and use a 27G needle to inject 50 μl of 2 mg/ml tamoxifen dissolved in corn oil. Although imaging can be carried out as early as postnatal day 2, we have found it most convenient to begin neonatal imaging at postnatal day 5 for reproducibility and ease of handling of neonatal pups.

**Figure 3.**
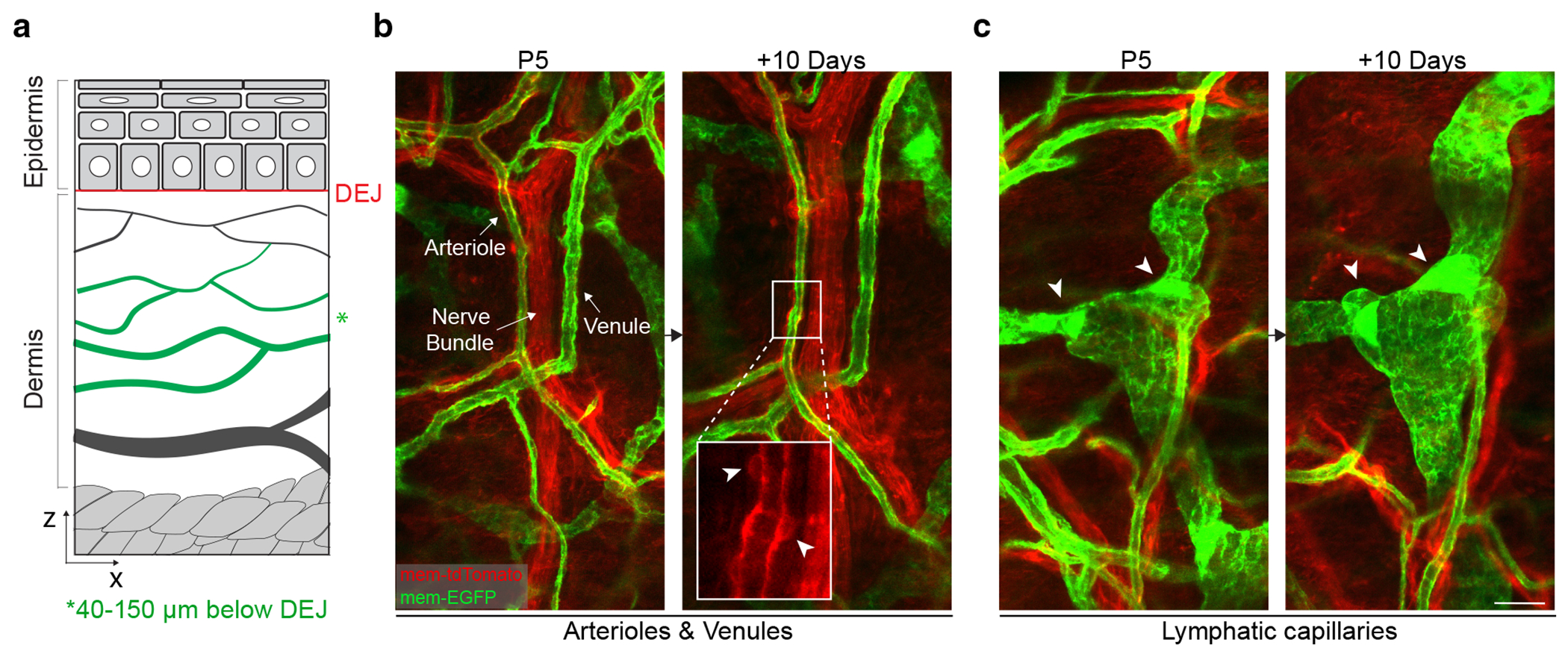
Identification of conserved vascular landmarks for longitudinal revisit imaging. (**a**) Schematic of neonatal paw skin under investigation. The intermediate plexus is highlighted in green. It is located ~;30—100 μm below the DEJ. (**b**) Arterioles and venules running parallel around nerve bundles provide a readily distinguishable conserved landmark for longitudinal revisits of the same vascular region. Inset: arteriolar SMC banding patterns visualized on the basis of mem-tdTomato signal. White arrowheads denote examples of arteriolar SMCs. (**c**) Alternatively, lymphatic vessels can be used as conserved landmarks. Lymphatic valves undergoing maturation are marked by white arrowheads. Bar = 40 μm. DEJ, dermal—epidermal junction; P5, postnatal day 5; SMC, smooth muscle cell.

Depending on the biological process under study, it is important to acquire a large enough vascular area that will be able to capture sufficient events. For example, to capture the remodeling dynamics of the maturing postnatal vasculature, we typically acquire regions of approximately 1.2 × 1.5 mm in area. Depending on the vessel subtype being studied, the upper and lower bounds of the Z-stack can be adjusted on the basis of the depth of the vessels of interest. We find that 2-μm Z-steps are sufficient to resolve overall vessel architecture, although smaller Z increments may be necessary if attempting to resolve finer structural detail. After the acquisition of the initial time point, longitudinal revisits can be carried out to track the remodeling dynamics of the maturing vasculature. To locate the same area of vasculature, we rely on identifying conserved landmarks within the tissue. We have found that the conserved organization of the arterioles and venules of the intermediate plexus that run parallel along the length of the paw provide reliable and consistent landmarks for longitudinal identification of previously imaged regions (Figure 3b). These structures are less prevalent than capillaries, making them easier to distinguish and are also subject to lower levels of remodeling during developmental stages, making them easier to identify between longitudinal time points. Note that arterioles and venules can be differentiated from each other on the basis of their smooth muscle cell (SMC) patterns, specifically the distinct banding pattern of arterial SMCs that are arranged on helical layers around the vessel. These banding patterns can be distinguished on the basis of the mem-tdTomato signal surrounding these vessels that is absent in venules (Figure 3b, inset). This same methodology can be used to distinguish arteries from veins. If utilizing a pan-EC driver such as *VECadCreER*, the patterns of the lymphatic vessels can also be used as reliable conserved landmarks for revisiting the same vascular areas (Figure 3c). Note that lymphatic vessels are subject to observable changes in their diameter during neonatal stages because this is an important developmental phase for the maturation of lymphatic valves (Figure 3c).

## ASSESSMENT OF HEMODYNAMICS AND VASCULAR BARRIER FUNCTION

**Figure 4.**
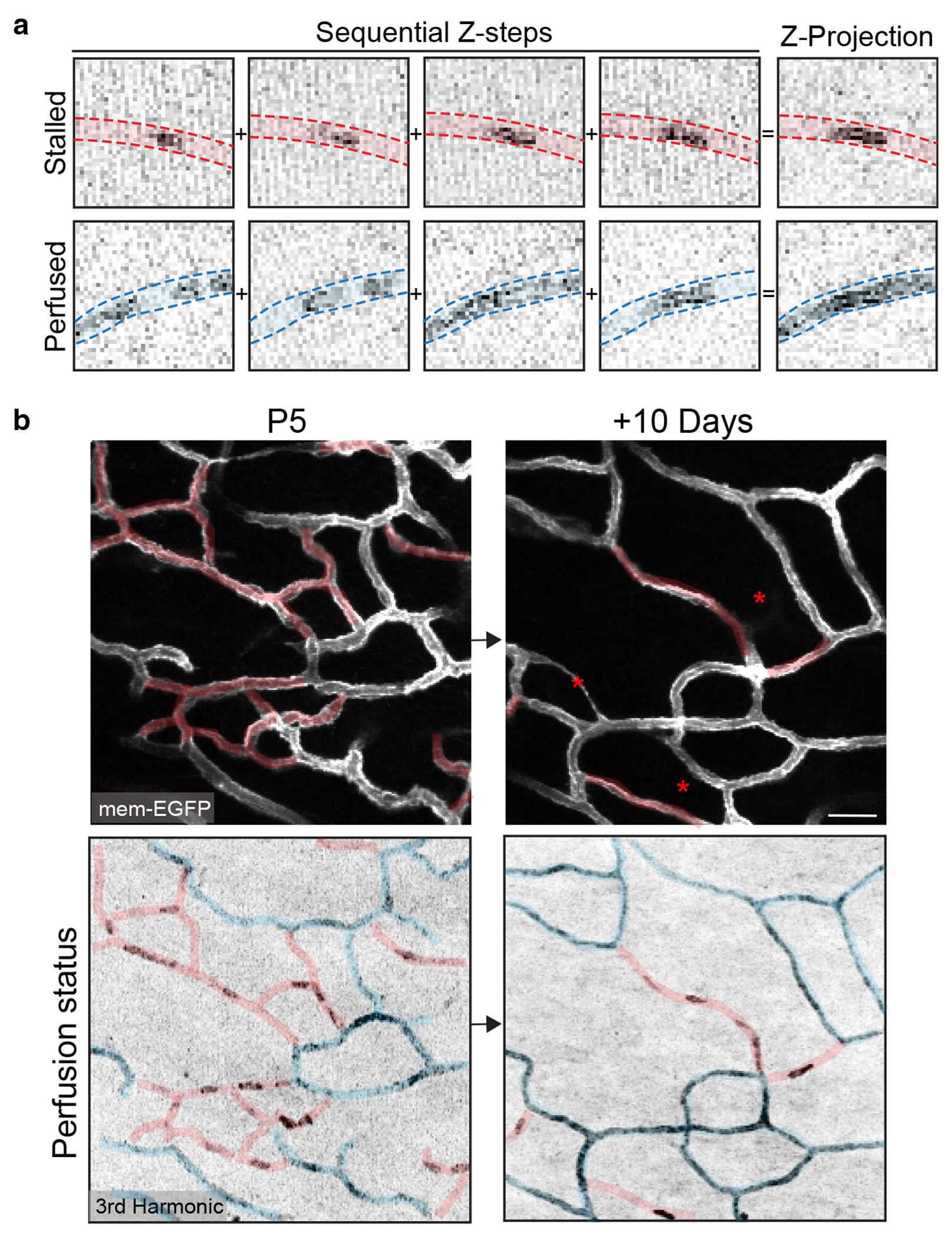
Visualization of hemodynamic parameters using third harmonic generation imaging. (**a**) The top panel shows an example of a series of Z-steps showing a single RBC that is stalled in a vessel segment. The bottom panel shows an example of a series of Z-steps showing movement of RBC in a perfused segment. The Z-projection of each series is shown to the right. Vessel locations are outlined with dotted lines. (**b**) An example of longitudinal tracking of perfusion status of neonatal capillaries tracked from P5 to P9. The top panel shows the vessel structures, whereas the bottom panel depicts the perfusion status. Perfused vessels are pseudocolored in blue, whereas nonperfused vessels are pseudocolored in red. Red asterisks denote location of regressed vessel segments. Bar = 20 μm. (**b**, adapted from Kam et al [2023], with permission from Elsevier). RBC, red blood cell; P5, postnatal day 5; P9, postnatal day 9.

Live imaging also presents the opportunity to assess vascular function in a variety of contexts such as during development, homeostasis, or disease states. Specifically, 2-photon microscopy can be used to measure hemodynamics as well as vascular permeability (Hartmann et al, 2021; Honkura et al, 2018). To measure hemodynamics, we have found success in leveraging third harmonic generation (THG) signal, a multi-photon imaging modality that enables the label-free visualization of red blood cells (RBCs) (Ferrer Ortas et al, 2023). THG imaging requires the use of a pulsed laser with a wide tuning range because visualization of THG signal requires excitation wavelengths above 1250 nm, with the strongest signal appreciated at 1275—1300 nm. The resulting emission signal can be detected in the 425—433 nm range. By performing acquisitions with fine Z increments (0.5-μm steps), this allows for the generation of Z-projections that provide a binary assessment of vessel perfusion status (Figure 4a). Perfused vessels exhibit a positive THG signal throughout the vessel segment because a result of flowing RBCs detected as the laser is scanning through the lumen of the vessel. In contrast, nonperfused vessels display either an absence of THG signal or the presence of single RBCs that are stalled within the vessel lumen (Figure 4a). Using this approach, we have previously determined the relationship between selective vessel regression and the optimization of network perfusion during neonatal development (Figure 4b) (Kam et al, 2023). Recently, Mesa et al (2025) utilized this method to understand the role of tissue resident macrophages for the maintenance of capillary perfusion in the context of aging skin. THG signals can also be utilized to measure more dynamic parameters of blood flow such as flow direction, speed, and flux through line scanning acquisition (Dietzel et al, 2014). This is an established methodology of measuring hemodynamics and has been described in detail by several articles (Ahn et al, 2020; Dietzel et al, 2014; Liu et al, 2020). Note that the appearance of the THG signal may differ depending on the scanning speed and detector abilities of specific microscope systems. Another consideration is that the same measurements can be performed using injection of fluorescently conjugated dextrans whereby the negative signal produced by cells within the bloodstream can be used to measure hemodynamics (Honkura et al, 2018).

Two-photon imaging can also be used to assess vascular barrier function. This can be achieved through injection of fluorescently conjugated dextran of specific molecular weights directly into the bloodstream through tail vein or retroorbital delivery. An elegant study from Honkura et al (2018) leveraged 2-photon microscopy of the mouse ear skin to provide a detailed understanding of cutaneous vascular permeability and its relationship to changes in vasotone and blood flow. In this study, the authors determined the identity of vessels that respond to VEGFA-induced permeability as well as the vasodilation and RBC velocity dynamics in response to VEGFA (Honkura et al, 2018). This study is an excellent resource for the systematic assessment of vessel identity and concurrent dynamic functions such as permeability and RBC parameters based on the visualization of injected fluorescent tracers (Honkura et al, 2018). To assess vessel barrier function, it is important to utilize the appropriate molecular-weight dextran for the specific level of permeability being assessed. For example, Honkura et al (2018) showed that in response to acute VEGFA164 local administration, a known stimulus of robust vessel permeability, this was sufficient to cause the rapid leakage of 2000 kDa FITC dextran out of cutaneous vessels. However, dependent upon the permeability stimulus being studied, lower-molecular-weight dextrans may be more appropriate to measure more subtle changes in vessel permeability. Consistent with the findings of Honkura et al (2018) in the ear skin vasculature, we have found that 70 kDa dextran is the smallest molecular weight that can be retained within the paw skin circulation when delivered into the bloodstream. Delivery of lower-molecular-weight dextrans such as 40 kDa results in an almost immediate and progressive leakage of dextran into the interstitial space.

## IMAGING THE PROCESS OF VASCULAR REPAIR AND REGENERATION

The endothelium constitutes a critical barrier that prevents the unregulated movement of fluids, macromolecules, and cells between the bloodstream and surrounding tissues (Claesson-Welsh et al, 2021). As such, the endothelium has evolved rapid and efficient mechanisms of repair and regeneration after injury (Evans et al, 2021). Although we have gained a significant understanding of the large-scale remodeling and signaling mechanisms involved in the vascular response to injury, there are still many open questions that remain regarding the spatiotemporal regulation of cellular behaviors that mediate vascular regeneration, particularly in the context of injuries that differ in scale and affected cell types.

**Figure 5.**
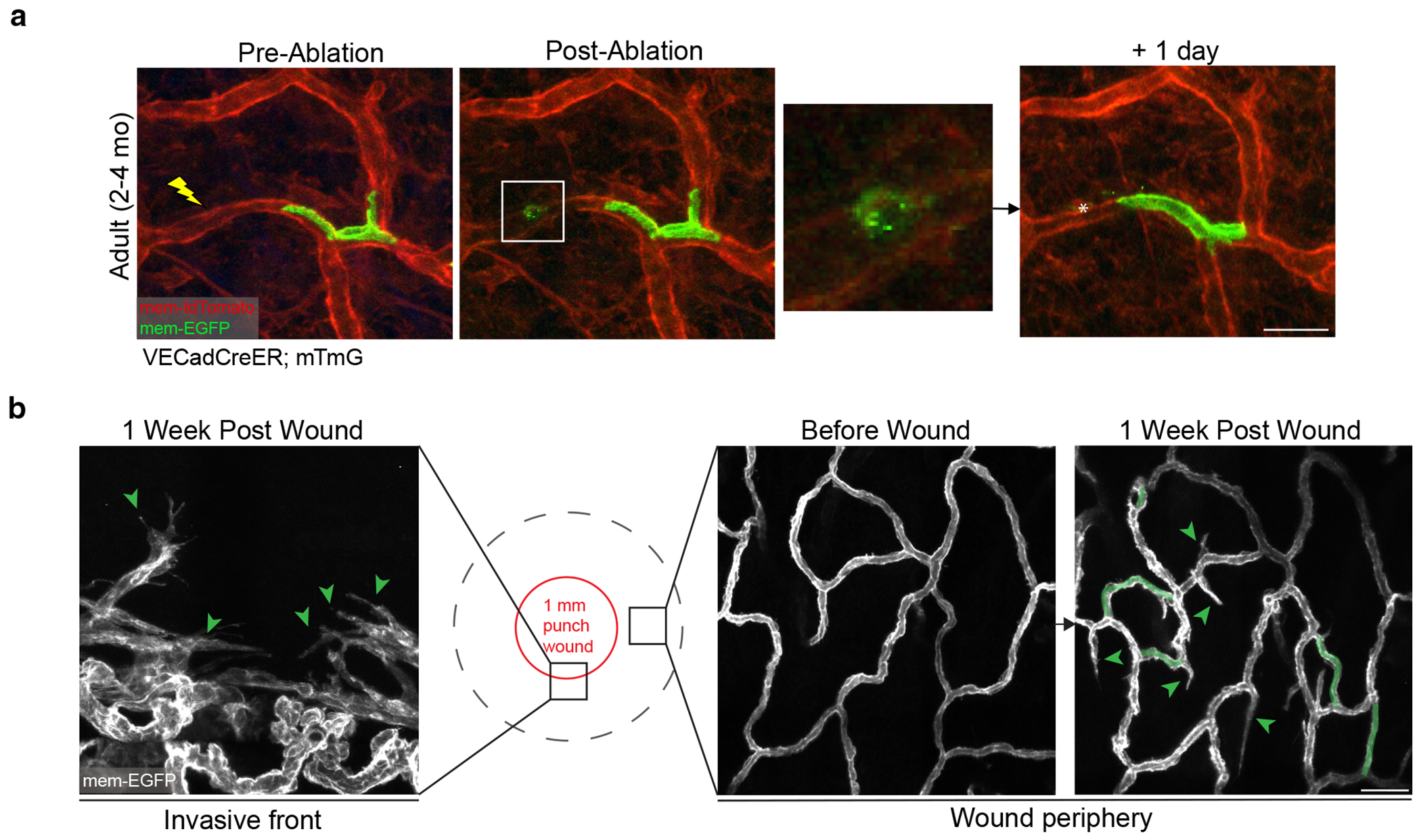
Injury models for studying vascular repair and regeneration. (**a**) A targeted laser ablation is performed adjacent to a single labeled adult EC, and its migratory response is recorded 1 day later. Lightning bolt denotes the site of ablation. An inset of the autofluorescent halo indicating success of the ablation is shown to the right of the postablation image. White asterisk denotes the ablated site 1 day later. Bar = 20 μm. (**b**) An example of longitudinal tracking of vascular regeneration after full-thickness punch wounding. One week after wounding, the robust angiogenic response can be visualized at the invasive front of the wound edge. In the wound periphery, examples of angiogenic sprouts (green arrowheads) and newly formed vessels (pseudocolored in green) can be detected. Bar = 40 μm. (**a**, adapted from Kam et al [2023], with permission from Elsevier). EC, endothelial cell.

To investigate the mechanisms of vascular repair and regeneration, we have employed a number of strategies to model vascular injury. One such method is targeted laser ablation, a versatile technique that we have used to model small-scale vascular injury (Kam et al, 2023). This process involves the use of pulsed femtosecond lasers, which are the standard lasers utilized in multiphoton microscopy, to inflict a concentrated pulse of laser power within a small focal volume that results in localized tissue damage to the targeted site (Figure 5a). The key advantage of targeted laser ablation is that it enables precise spatiotemporal control of the injury process. This methodology can be used to ablate small numbers of cells in specific locations within the tissue to then monitor the repair process. Before performing targeted laser ablation, one should first select the region of interest (ROI) and perform imaging of this region to capture the state of the vascular network prior to the laser injury. After this acquisition, a small ROI should be selected over the site of the desired ablation. To induce focal injury or single-cell ablation, we recommend selecting a ROI of 2 × 2 μm in size. The laser can then be tuned to the appropriate wavelength and power level depending on the make and manufacturer of said laser. We have had success performing ablations at 850-nm wavelength and 15% laser power (on the basis of ~2.5 mW power output). Application of live scanning for several seconds will then allow for the ablation to occur. After live scanning, an acquisition of the full field of view will allow for confirmation of the ablation procedure. An autofluorescent halo should be detectable at the ablation site that indicates the successful induction of thermal damage (Figure 5a). It is important to note that if this halo is not detected, it is possible that the scanned area has been photobleached rather than afflicted with thermal damage. After ablation, time-lapse imaging can be performed to capture the immediate effects of the ablation, followed by subsequent longitudinal revisits at desired time points to resolve the longer-term effects.

To model large-scale ablation of ECs, we have also employed the use of a genetic ablation model that relies upon the Cre dependent expression of diphtheria toxin (*Rosa26-DTA*) (Voehringer et al, 2008). When combined with an EC-specific Cre driver, this enables tamoxifen inducible expression of diphtheria toxin A (DTA) in ECs, resulting to widespread cell ablation. One caveat of this approach is that because induction of DTA expression is Cre dependent, this model is no longer compatible with Cre-dependent fluorescent reporters. As such, a constitutive reporter of ECs is required. We have utilized the *VECad-mTnG* model (also referred to as *Cdh5-mTnG*) for this purpose that constitutively expresses membrane-bound tdTomato and nuclear H2B-EGFP, allowing for visualization of vessel architecture and all EC nuclei (Jeong et al, 2017). Another important consideration for utilizing this model is the dosage of tamoxifen that is administered because systemic ablation of too large of a fraction of ECs can lead to hemorrhage and death of the animal. We have found that a dose of 10 mg/kg of tamoxifen delivered intraperitoneally is sufficient to ablate ~25% of the skin EC network while maintaining the overall survival and health of the animal. The majority of cellular death occurs within the first 6 days after tamoxifen administration, which is then followed by the gradual recovery of the endothelial network (Kam et al, 2023). This model has proven useful for the interrogation of the mechanisms underlying vascular maintenance but could also be applied to studies seeking to understand the regulation of tissue-resident endothelial progenitors during the process of regeneration.

Longitudinal imaging also presents the opportunity to understand the process of wound vascularization. An elegant study from Chong et al (2017) leveraged longitudinal imaging of ear skin to identify the development of tortuous micro-vessels in response to wounding as well as the fact that the majority of wound-induced angiogenesis arises from tortuous vessels. We have confirmed similar observations in wounding of paw skin and utilized this approach to track the process of vascular maturation after the initial wave of angiogenesis induced by wounding injury (Kam et al, 2023). To induce this injury, we perform a full-thickness wound using a 1-mm punch biopsy positioned in the center of the hairless area of the mouse hind paw. Wounded paw skin provides a tractable model to study adult angiogenesis because we are able to observe the wave of new vessel growth that occurs at the invasive front of the wound edge as well as nascent sprouting angiogenesis that occurs in the wound periphery (Figure 5b). This presents an opportunity to study the spatial zonation of wound revascularization as well as the dynamic cellular behaviors that drive the regeneration of the skin vascular network.

## FUTURE OUTLOOK

The ability to track vascular architecture and EC behaviors also provides an opportunity to deepen our understanding of skin diseases that are caused by or involve alterations to the cutaneous vasculature. For example, intravital imaging would allow for the investigation of inflammatory skin diseases with known vascular involvement such as psoriasis, rosacea, and systemic sclerosis (Kulkarni et al, 2020; Leong et al, 2005; Pattanaik et al, 2011). This approach could be utilized to study the changes in vascular architecture and EC behaviors as well as model the interactions of various immune cell types with the cutaneous vasculature. Furthermore, through the use of genetic models, longitudinal imaging provides an opportunity to study the mechanisms underlying the development of vascular malformations, developmental anomalies that are associated with specific genetic and somatic sequence variants, many of which are known to present in the skin (Nguyen et al, 2020), for example, the genetic vascular disorder, hereditary hemorrhagic telangiectasia types I and II, caused by inherited variants in the *ENG* and *ACVRL1* genes, respectively (Abdalla and Letarte, 2006). This disease leads to the development of arteriovenous malformations (AVMs) throughout the body, including smaller AVMs referred to as telangiectasias that present in the skin (Ortman and Ortolani, 2024). Mouse models harboring EC conditional deletion of *ENG* and *ACVRL1* faithfully recapitulate various aspects of the human disease, providing an opportunity to understand the cellular mechanisms underlying the development of these vascular malformations (Han et al, 2014; Tual-Chalot et al, 2020). Similarly, longitudinal imaging may also provide opportunities to investigate the pathogenesis of vascular anomalies caused by somatic variants. Diseases such as Klippel—Trénaunay syndrome and Sturge—Weber syndrome are caused by somatic variants in *PIK3CA* and *GNAQ* genes, respectively, leading to blood vessel abnormalities known to present in the skin (Nguyen et al, 2020). Mouse models harboring patient-specific variants in these genes present a unique ability to track the clonal lineage and specific vascular remodeling dynamics that drive the malformations observed in these diseases (Kobialka et al, 2022; Smits et al, 2025). In summary, longitudinal imaging of skin vasculature is a useful and versatile technique that will allow us to deepen our understanding of mammalian skin and vascular biology.

## Supplementary Material

1

Supplementary material is linked to this paper. Teaching slides are available as supplementary material.

## Abbreviations:

AVM: arteriovenous malformation
DTA: diphtheria toxin A
EC: endothelial cell
IG: intragastric
IP: intraperitoneal
RBC: red blood cell
ROI: region of interest
SMC: smooth muscle cell
THG: third harmonic generation
