# Supplementary material for: Live imaging of vascular dynamics in mouse skin
Source: J Invest Dermatol. Author manuscript; Available in PMC 2026 Jul 13. (PMC13364158; doi:10.1016/j.jid.2025.12.031)
Supplement: 1 [file NIHMS2189428-supplement-1.pptx]

## Slide 1
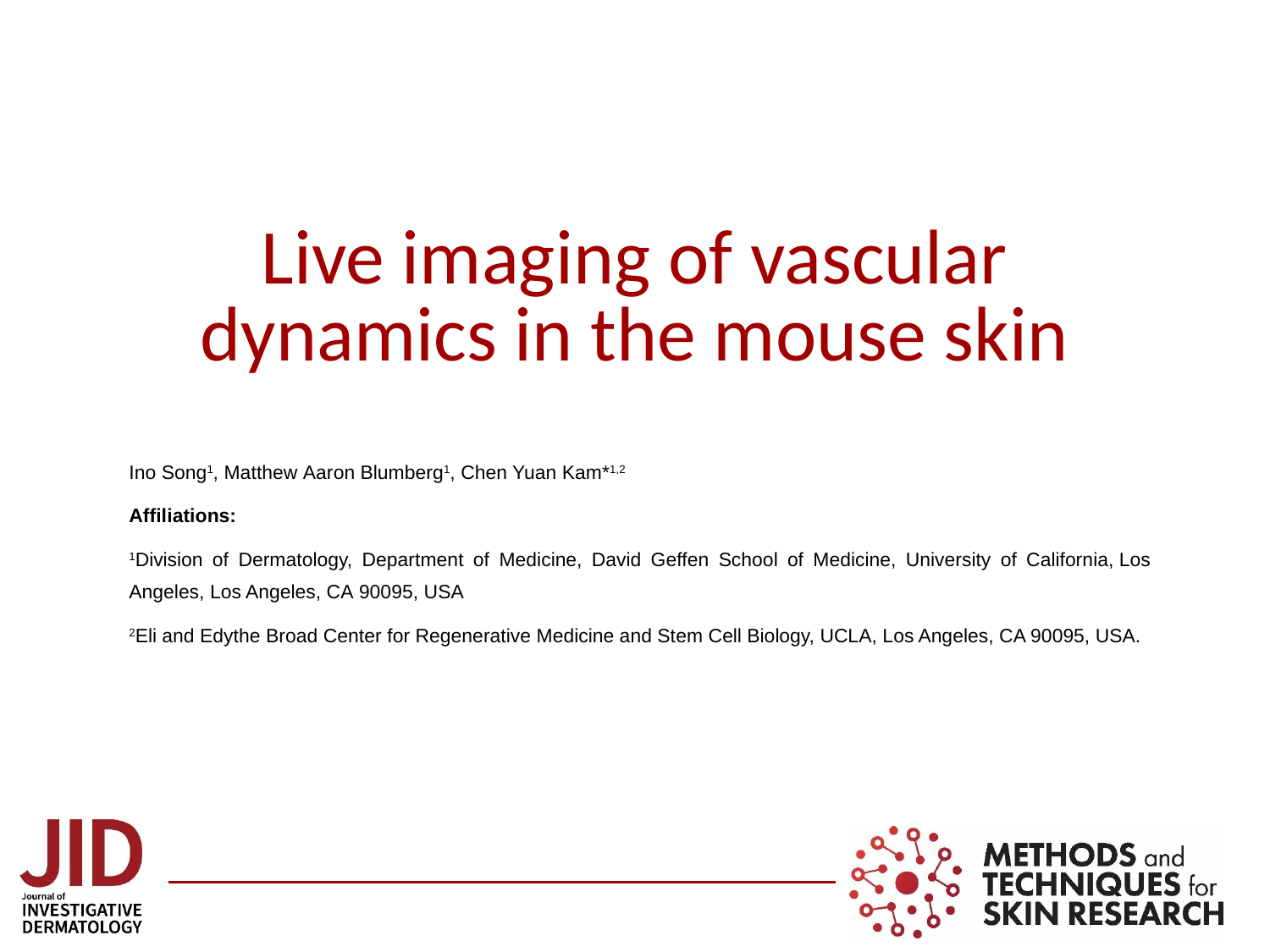

# Live imaging of vascular dynamics in the mouse skin
Ino Song1, Matthew Aaron Blumberg1, Chen Yuan Kam*1,2
Affiliations:
1Division of Dermatology, Department of Medicine, David Geffen School of Medicine, University of California, Los Angeles, Los Angeles, CA 90095, USA
2Eli and Edythe Broad Center for Regenerative Medicine and Stem Cell Biology, UCLA, Los Angeles, CA 90095, USA.

## Slide 2
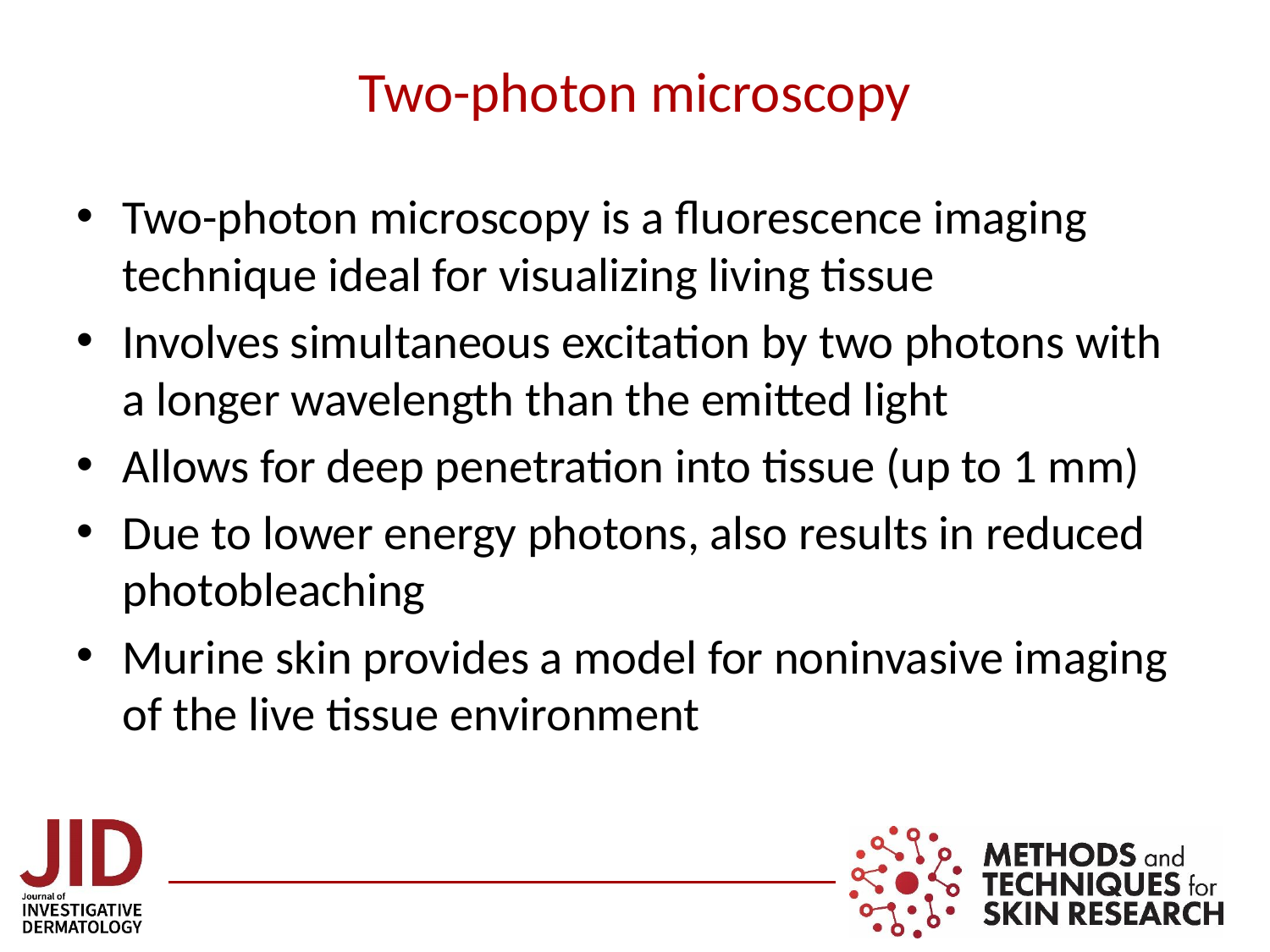

# Two-photon microscopy
Two-photon microscopy is a fluorescence imaging technique ideal for visualizing living tissue
Involves simultaneous excitation by two photons with a longer wavelength than the emitted light
Allows for deep penetration into tissue (up to 1 mm)
Due to lower energy photons, also results in reduced photobleaching
Murine skin provides a model for noninvasive imaging of the live tissue environment

## Slide 3
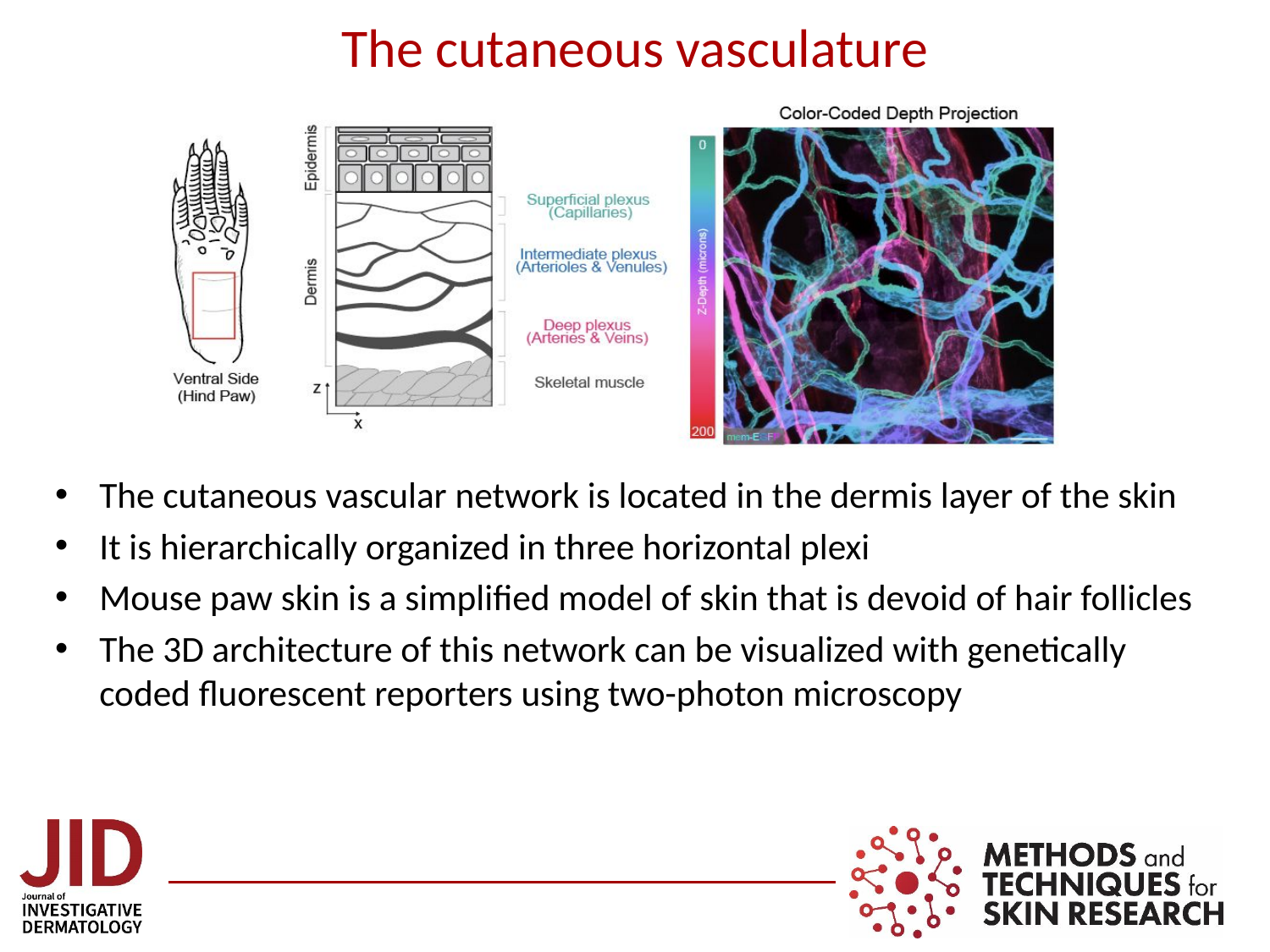

# The cutaneous vasculature
The cutaneous vascular network is located in the dermis layer of the skin
It is hierarchically organized in three horizontal plexi
Mouse paw skin is a simplified model of skin that is devoid of hair follicles
The 3D architecture of this network can be visualized with genetically coded fluorescent reporters using two-photon microscopy

## Slide 4
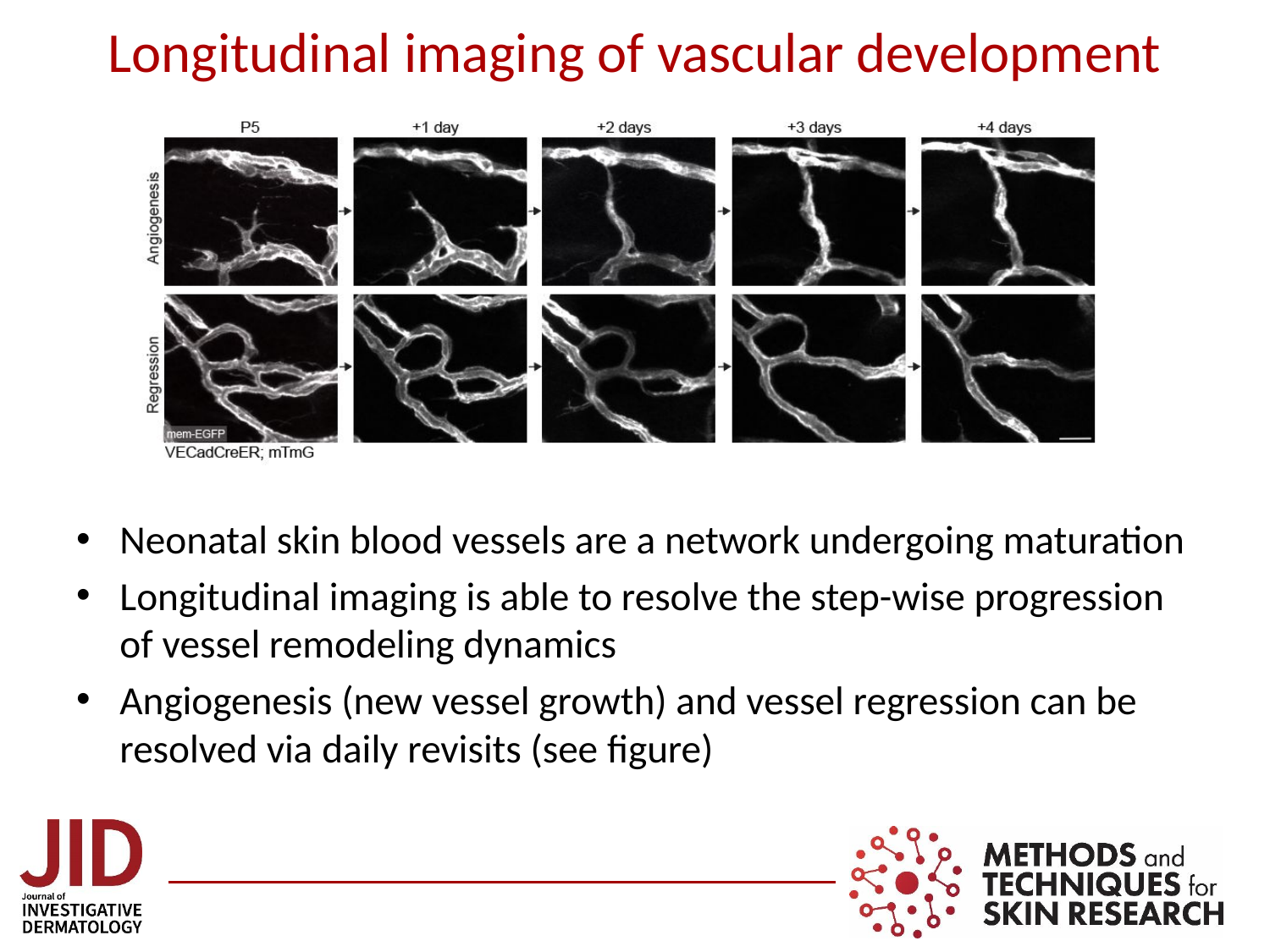

# Longitudinal imaging of vascular development
Neonatal skin blood vessels are a network undergoing maturation
Longitudinal imaging is able to resolve the step-wise progression of vessel remodeling dynamics
Angiogenesis (new vessel growth) and vessel regression can be resolved via daily revisits (see figure)

## Slide 5
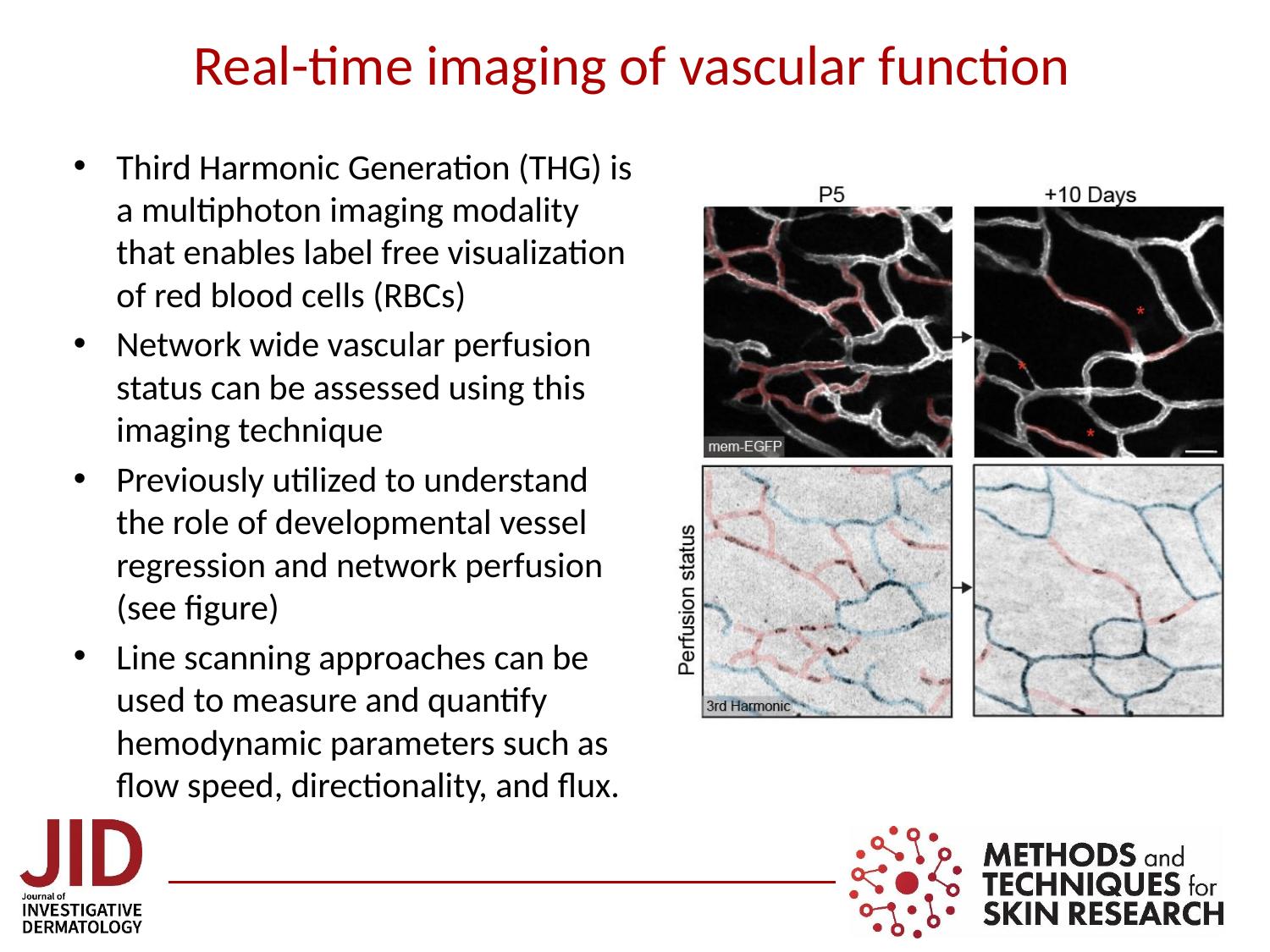

# Real-time imaging of vascular function
Third Harmonic Generation (THG) is a multiphoton imaging modality that enables label free visualization of red blood cells (RBCs)
Network wide vascular perfusion status can be assessed using this imaging technique
Previously utilized to understand the role of developmental vessel regression and network perfusion (see figure)
Line scanning approaches can be used to measure and quantify hemodynamic parameters such as flow speed, directionality, and flux.

## Slide 6
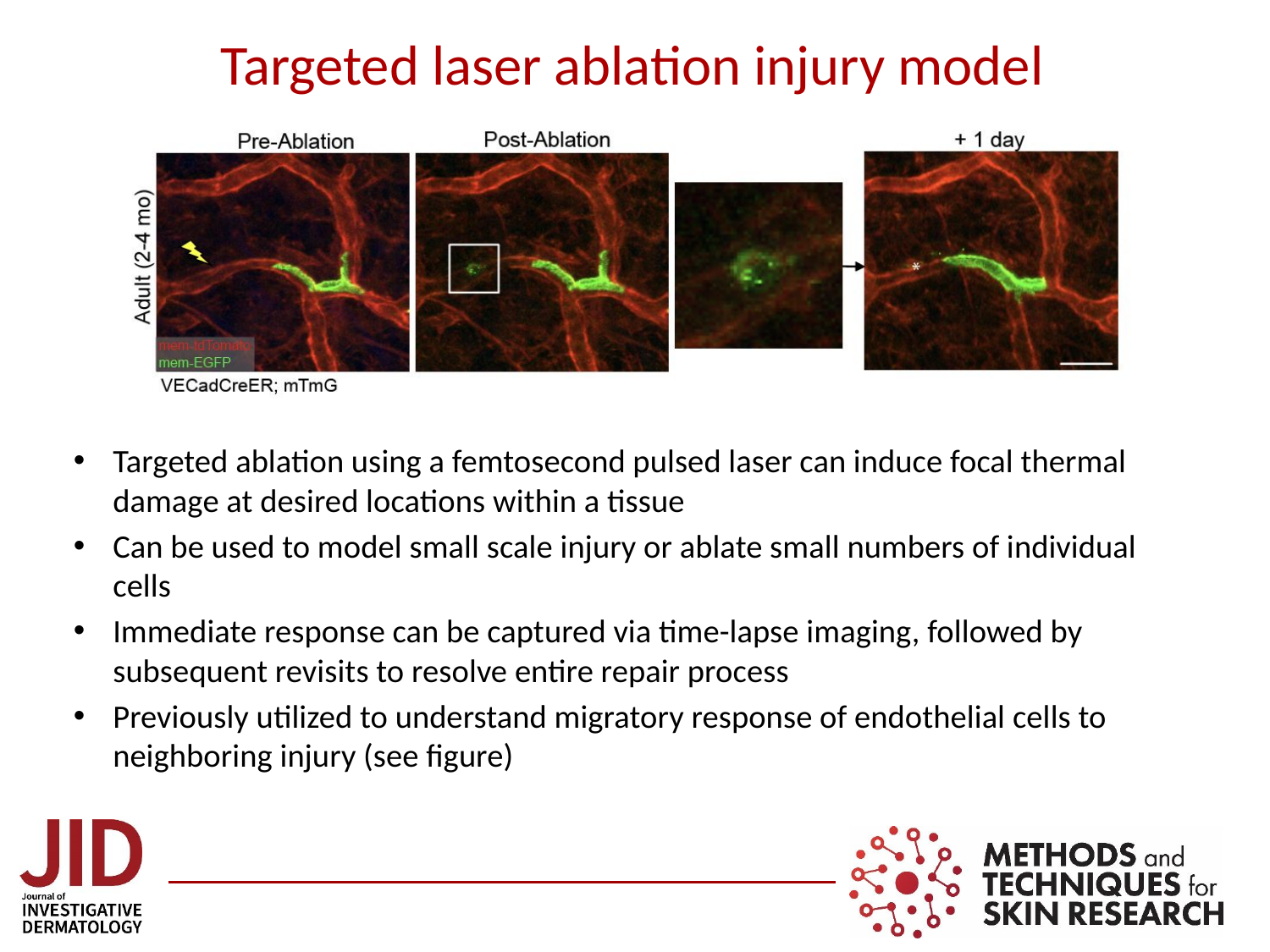

Targeted laser ablation injury model
Targeted ablation using a femtosecond pulsed laser can induce focal thermal damage at desired locations within a tissue
Can be used to model small scale injury or ablate small numbers of individual cells
Immediate response can be captured via time-lapse imaging, followed by subsequent revisits to resolve entire repair process
Previously utilized to understand migratory response of endothelial cells to neighboring injury (see figure)

## Slide 7
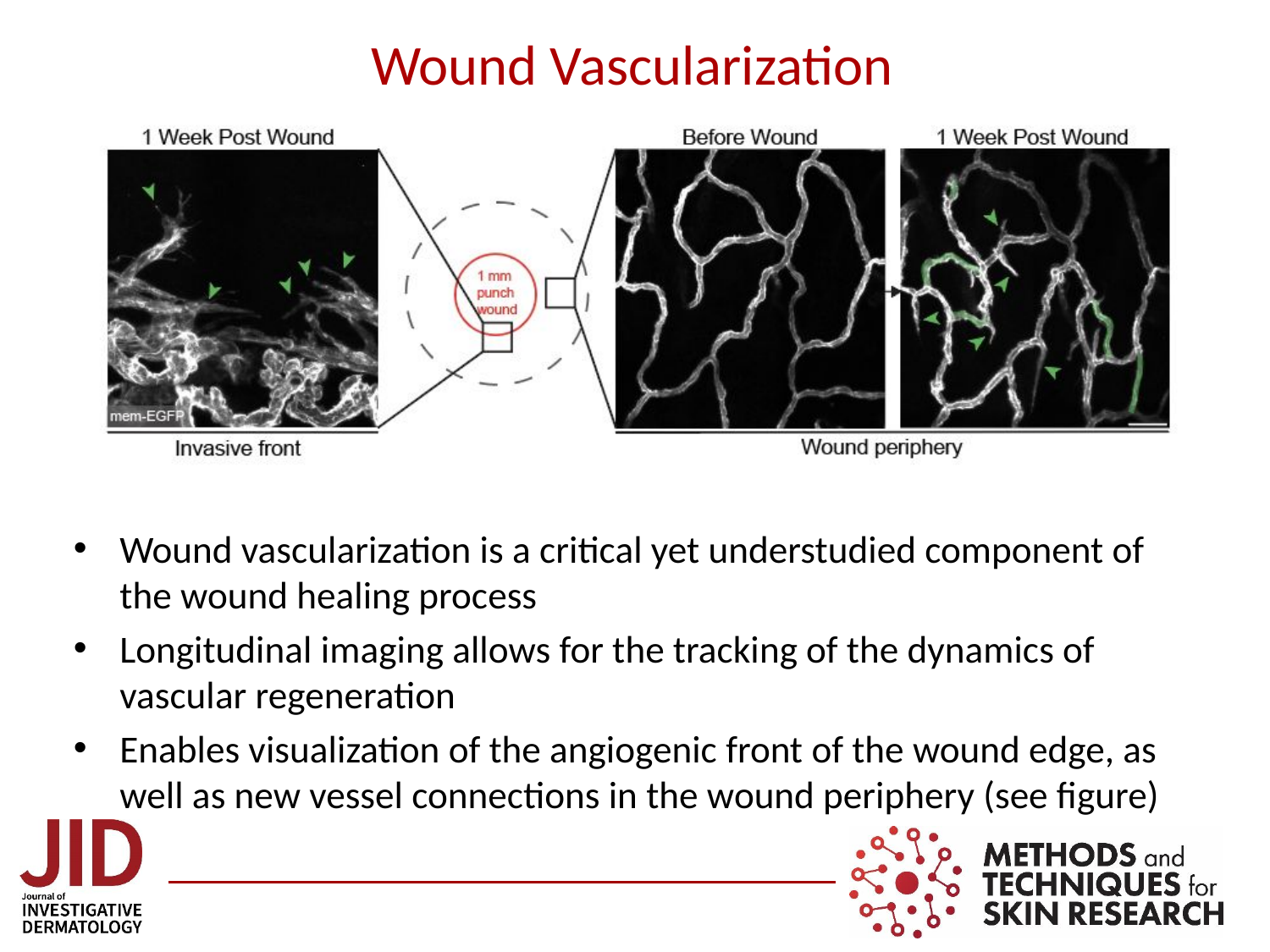

Wound Vascularization
Wound vascularization is a critical yet understudied component of the wound healing process
Longitudinal imaging allows for the tracking of the dynamics of vascular regeneration
Enables visualization of the angiogenic front of the wound edge, as well as new vessel connections in the wound periphery (see figure)

## Slide 8
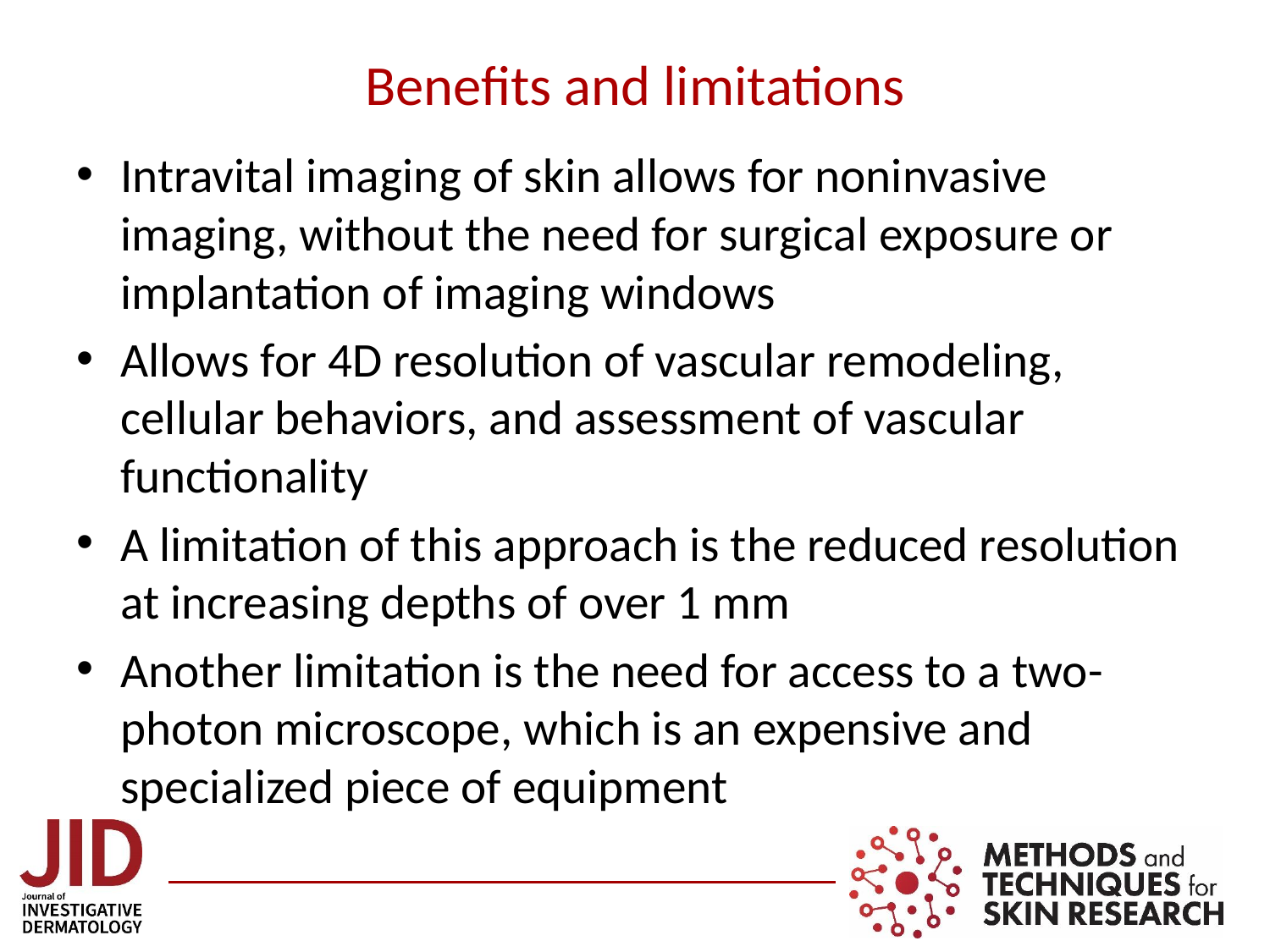

# Benefits and limitations
Intravital imaging of skin allows for noninvasive imaging, without the need for surgical exposure or implantation of imaging windows
Allows for 4D resolution of vascular remodeling, cellular behaviors, and assessment of vascular functionality
A limitation of this approach is the reduced resolution at increasing depths of over 1 mm
Another limitation is the need for access to a two-photon microscope, which is an expensive and specialized piece of equipment

## Slide 9
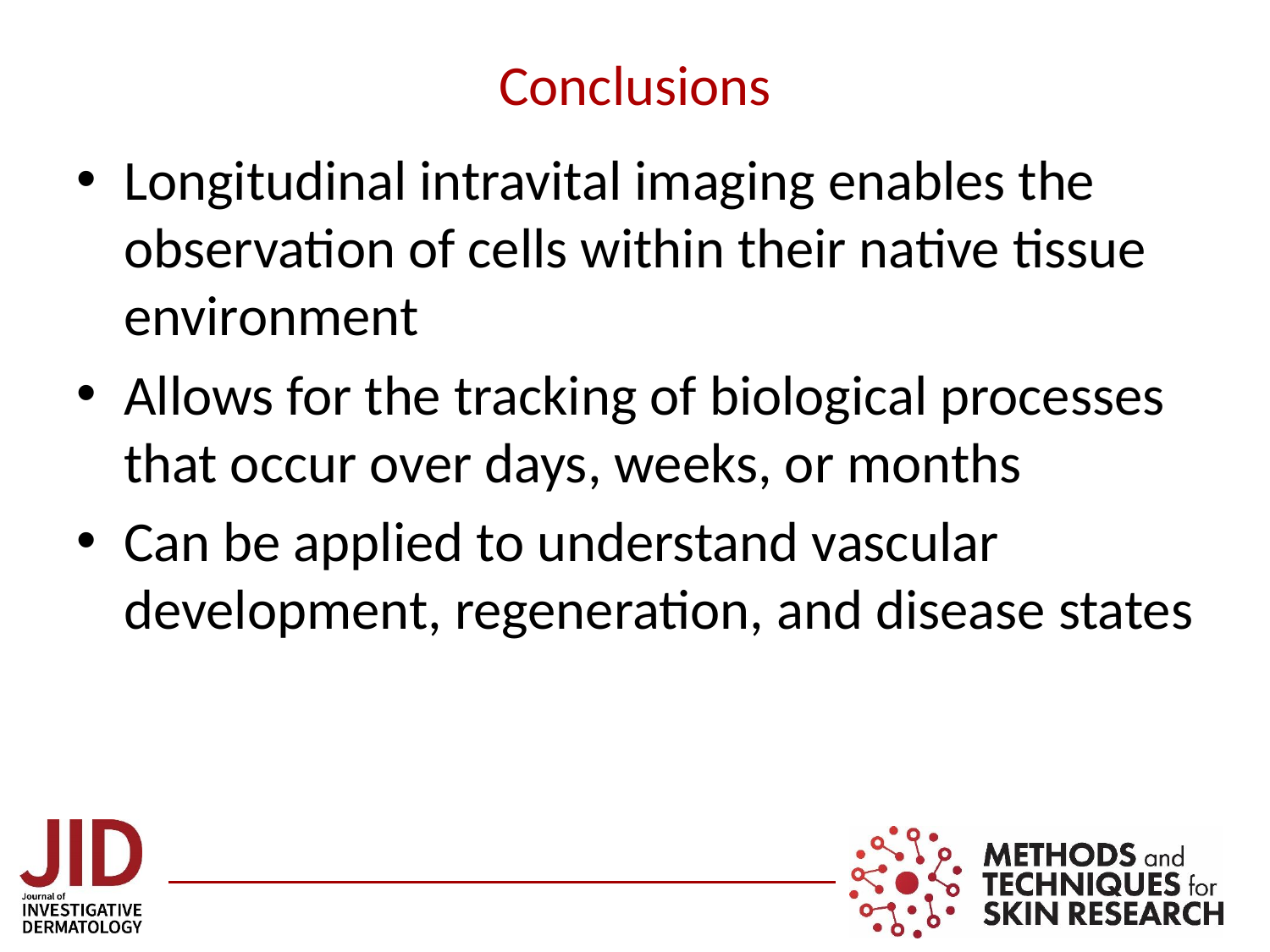

# Conclusions
Longitudinal intravital imaging enables the observation of cells within their native tissue environment
Allows for the tracking of biological processes that occur over days, weeks, or months
Can be applied to understand vascular development, regeneration, and disease states
